# The effectiveness of the National Veterans Summer Sports Clinic for veterans with probable posttraumatic stress disorder

**DOI:** 10.3389/fpsyg.2023.1207633

**Published:** 2023-07-10

**Authors:** Kristen H. Walter, Nicholas P. Otis, Michal Kalli Hose, Kathleen M. Ober, Lisa H. Glassman

**Affiliations:** ^1^Health and Behavioral Sciences, Naval Health Research Center, San Diego, CA, United States; ^2^VA San Diego Healthcare System, San Diego, CA, United States; ^3^Leidos, Inc., San Diego, CA, United States

**Keywords:** physical activity, outdoor recreation programs, adaptive sports, veterans, PTSD, nature exposure, outdoor activity

## Abstract

**Introduction:**

Recreational and adaptive sports programs may be beneficial therapeutic interventions for improving psychological outcomes among veterans and service members with posttraumatic stress disorder (PTSD) because they provide opportunities for exercise, socialization, respite, and time outdoors. Although there are an increasing number of sports programs for veterans and service members with PTSD, data evaluating the outcomes of such programs are limited.

**Materials and methods:**

The sample included 74 U.S. veterans who participated in the National Veterans Summer Sports Clinic (NVSSC), an annual, week-long adaptive sports program in San Diego, California. Participants were categorized into two subgroups: those who met criteria for probable PTSD using the PTSD Checklist for DSM-5 (*n* = 20) and those who did not (*n* = 54). Participants completed self-report assessments before and after each daily activity, before and after the program, and 3 months following program completion.

**Results:**

Over the course of NVSSC program participation, the diagnostic subgroups (probable PTSD vs. no PTSD) did not significantly differ on changes in depression, positive affect, negative affect, or insomnia. Compared to those without PTSD, veterans with PTSD experienced greater reduction in generalized anxiety during the program (*MD* = −3.07, *p* = 0.034). Veterans with PTSD also experienced significant improvements in PTSD symptoms at postprogram (*MD* = −23.76, *p* < 0.001). For both groups, significant benefits were shown during the program but rebounded by the 3-month follow-up. Over the course of each daily activity, positive affect (*MD* = 2.71, *p* < 0.001) and depression/anxiety scores significantly decreased (*MD* = −0.75, *p* < 0.001), with no differences between PTSD diagnostic groups across time (*p*s = 0.714 and 0.961, respectively).

**Conclusion:**

Veterans with and without PTSD benefited from participation in the NVSSC. Participants with probable PTSD experienced greater improvements in generalized anxiety at postprogram only; there were no other significant differences between the two groups at postprogram or at 3-month follow-up. In line with prior research, benefits for those with PTSD were lost by 3-month follow-up, suggesting that regular engagement in recreational and adaptive sports may be necessary to sustain psychological health improvements.

## Introduction

The availability of recreational and adaptive sports programs for veterans and military service members has increased dramatically over the last decade. Mounting evidence supports the positive impact on psychological symptoms, well-being, and other health-related outcomes (Davis-Berman et al., 2018; Greer and Vin-Raviv, 2019; Walker et al., 2020) as well as the psychological benefits of exercise among both community (e.g., see Mikkelsen et al., 2017 for a review) and psychiatric samples (e.g., Rethorst et al., 2013; Schuch et al., 2016). Recreational and adaptive programs center on participant engagement in physical activities (e.g., sports, swimming, fishing, canoeing) and create adaptations for varying physical and psychological abilities. These adaptations may include modifying equipment such as bicycles or skis, using wheelchairs, or allowing caregiver participation/support. Recreational and adaptive programs are particularly valuable because they encourage physical exercise, often in a natural environment and with social interaction, factors that have been linked to improvements in mental health, quality of life, and functioning (e.g., Greer and Vin-Raviv, 2019; Walter et al., 2019; Wheeler et al., 2020; Walter et al., 2021; Sidiropoulos et al., 2022).

The National Veterans Summer Sports Clinic (NVSSC) and Winter Sports Clinic (NVWSC) are examples of recreational and adaptive sports programs offered to veterans with physical (e.g., limb amputation) or psychological [e.g., posttraumatic stress disorder (PTSD)] injuries, and preliminary data suggest that both programs have positive impacts on the mental health of veterans who attend. A recent study examined NVSSC outcomes (*N* = 74; Walter et al., 2021) and found that immediately following the week-long program, veteran participants reported improvements in depression, anxiety, social functioning, and negative and positive affect. However, those gains were not maintained at 3-month follow-up (Walter et al., 2021). A separate study examining outcomes following two recreational and adaptive sports clinics for veterans (NVWSC and the National Veterans Wheelchair Games; *N* = 132) reported improvements in quality of life and self-esteem for attendees (Sporner et al., 2009). Increasing access to nature-based physical activities for veterans and service members with psychological and physical injuries represents an avenue for improving mental health, and such activities may also be less likely to trigger fears of stigma and treatment avoidance, which can be concerns for these populations (e.g., Forsyth et al., 2020).

Veterans and service members with PTSD may particularly benefit from engagement in adaptive and recreational programs. PTSD is one of the most common mental health conditions among veterans and service members (Fulton et al., 2015; Thomas et al., 2017) and significantly impacts the individual, their family, and greater community (e.g., Taft et al., 2011; Creech and Misca, 2017). Although the U.S. Department of Veterans Affairs (VA) has provided infrastructure and extensive resources devoted to evidence-based PTSD treatments, there are veterans who do not respond to these treatments or who are reticent to seek traditional psychological interventions (e.g., Stecker et al., 2013; Hoge et al., 2014; Steenkamp et al., 2015), highlighting the need for additional treatment options. Although research is limited, two recent literature reviews (Davis-Berman et al., 2018; Greer and Vin-Raviv, 2019) found that outdoor interventions may have positive psychological and psychosocial benefits and improve quality of life among veterans with PTSD. However, both reviews stressed that these findings should be interpreted with caution, citing the weak methodological quality of existing studies.

Recent studies have continued to support the short-term positive effects of these programs on psychological and psychosocial functioning among veterans and service members with PTSD, but they suffer from similar methodological limitations (e.g., Townsend et al., 2018; Walter et al., 2019; Wheeler et al., 2020; Hooker et al., 2022). First, few studies used control groups, which limits understanding program impact compared with how participants might have fared without intervention. Additionally, most studies used small sample sizes, and across studies, significant variability exists in program length or type of recreational activities offered, thereby limiting generalizability. Lastly, few studies included a follow-up to determine whether benefits persisted over time. Although there is a larger body of research supporting the immediate benefits of outdoor physical activities among broader populations (e.g., Thompson Coon et al., 2011; Walter et al., 2019, 2021), the long-term impact of these programs remains unclear. Most studies have not examined outcomes beyond a few weeks after program conclusion, but more recent studies have extended follow-up assessments to 3–9 months (e.g., Townsend et al., 2018; Walter et al., 2019; Hooker et al., 2022). Though scant, longitudinal data suggest that many psychological health outcomes are not sustained over time (e.g., Townsend et al., 2018; Walter et al., 2019; Hooker et al., 2022). As such, it is critical that research improve upon the methodological limitations of the existing research to understand the outcomes of these programs and the duration of effects for veterans and service members with PTSD.

The current study sought to extend the findings of Walter et al. (2021) by examining PTSD outcomes following the annual NVSSC program, and by comparing other psychological outcomes between veteran participants with and without probable PTSD. The goal of this naturalistic study was to evaluate NVSSC program outcomes among veterans with probable PTSD, without changing the intended NVSSC population or program structure. The parent study (Walter et al., 2021) found that overall, participating veterans experienced significant pre- to postprogram changes in depression, generalized anxiety, social functioning, and positive and negative affect. This study also builds on the methodological limitations of the existing literature by incorporating a comparison group (i.e., probable PTSD vs. no PTSD), using well-validated measures commonly used with the military population, and examining immediate, short-term, and longer-term outcomes. Exploring outcomes among veterans and service members with PTSD is an important research endeavor, given that recreational and adaptive sports programs are often provided to these specific populations; however, data evaluating such program outcomes are limited. Furthermore, results can inform the delivery of recreational and adaptive sports programs by identifying who benefits most from these interventions, what outcomes can be expected, and how these interventions may work in tandem with traditional treatment approaches.

## Materials and methods

### Program

The NVSSC is an annual, week-long adaptive sports program for veterans with physical and psychological injuries that is provided by the VA. Veterans from across the United States travel to San Diego, California, to participate in a different recreational activity each day over 5 days, including cycling, surfing, sailing, kayaking, and archery/pickleball. Recreational activity instruction is provided by trained instructors and volunteers, and equipment and adaptations are made based on physical and/or psychological need. Veterans are assigned to teams of approximately 10 participants for the duration of the week. Data analyzed for this naturalistic study were collected from participants in the 2017 and 2018 NVSSC programs.

### Participants

To be eligible for the NVSSC, U.S. veterans were required to have a psychological (e.g., PTSD, depression) or physical (e.g., amputation, traumatic brain injury) diagnosis as a reason for program referral. There were several conditions for which veterans were not granted medical clearance such as significant cardiovascular risk. Otherwise, there were no additional exclusion criteria for the parent study, and data from all 74 participants were analyzed in both studies (see Walter et al., 2021). In the current analysis, participants were categorized into two groups *a posteriori* based on DSM-5 diagnostic criteria and data from the preprogram Life Events Checklist (LEC; Weathers et al., 2013a) and PTSD Checklist for DSM-5 (PCL-5; Weathers et al., 2013b): probable PTSD (*n* = 20) and no PTSD (*n* = 54). Importantly, 100% of the probable PTSD and 57% of the non-PTSD subgroups reported concurrent mental health treatment (e.g., medication, psychotherapy).

### Procedure

Participants were recruited at the host hotel during the NVSSC orientation meeting and clinic exposition. Veterans interested in study participation provided informed consent and completed a preprogram assessment; those who did not consent took part in the program as offered. Veterans then participated in a different recreational activity each day over 5 days as part of the larger team to which they were assigned. Brief self-report assessments were completed before and after each daily recreation activity. A postprogram assessment was completed on the final day of the program. Lastly, participants completed a follow-up assessment *via* phone 3 months after the NVSSC. The 3-month assessment was selected as a sufficient timeframe to determine whether gains were maintained after participants returned to their normal lives without risking significant participant attrition. All study procedures were approved by the VA San Diego Healthcare System Institutional Review Board.

### Measures

Posttraumatic stress disorder symptom severity in the past month was evaluated with the LEC (Weathers et al., 2013a) and PCL-5 (Weathers et al., 2013b). The PCL-5 contains 20 questions rated on a 0–4 scale, with total scores ranging from 0 to 80 (higher scores indicate greater PTSD severity). In this study, internal consistency was *α* = 0.87–0.97 across time points. Probable PTSD diagnosis was determined by matching Diagnostic and Statistical Manual of Mental Disorders, Fifth Edition (DSM-5; American Psychiatric Association, 2013) criteria with responses on the LEC (criterion A) and PCL-5. Specifically, probable PTSD criteria were met if participants reported a Criterion A traumatic event on the LEC-5 and indicated a rating of 2 or higher (i.e., “moderately” to “extremely” bothersome) on at least one cluster B symptom, one cluster C symptom, two cluster D symptoms, and two cluster E symptoms on the PCL-5.

Depression symptom severity during the past 2 weeks was measured with the 8-item Patient Health Questionnaire (PHQ-8; Kroenke et al., 2009b). The 8-item version of the PHQ (i.e., version without the suicide item) was selected due to our inability to adequately manage suicide risk in this naturalistic setting. PHQ-8 items are summed to a total severity score (range 0–24; higher scores reflect greater severity). Across assessment points, internal consistency for the PHQ-8 was *α* = 0.86–0.89. Depression/anxiety symptoms were also measured before and after each activity with the 4-item Patient Health Questionnaire (PHQ-4; Kroenke et al., 2009a). Like the PHQ-8, PHQ-4 items are summed to a total score (scale range 0–12; *α* = 0.72–0.84 across activity).

Generalized anxiety symptoms were evaluated with the Generalized Anxiety Disorder-7 (GAD-7; Spitzer et al., 2006). The frequency of each symptom in the past 2 weeks was rated on a 4-point scale, which is summed to create an overall score ranging from 0 to 21 (higher scores indicate greater generalized anxiety severity). Internal consistency was *α* = 0.91 at all-time points.

Insomnia was examined with the Insomnia Severity Index (ISI; Morin and Barlow, 1993). The ISI is a 7-item, self-report measure that can be summed to a total severity score (range 0–28; higher scores indicate greater insomnia severity). Across time points, internal consistency for the ISI was *α* = 0.90–0.93.

Affect was assessed with the Positive and Negative Affect Schedule (PANAS; Watson et al., 1988). The PANAS is a 20-item, self-report measure consisting of 10 positive and 10 negative emotions experienced over the past several hours. The Positive Affect Schedule (PAS) and Negative Affect Schedule (NAS) are each summed to create subscales, with higher scores (10–50) indicating greater intensity of affect. Cronbach’s alphas were *α* = 0.89–0.92 and *α* = 0.86–0.91 for the PAS and NAS subscales, respectively, across longitudinal time points. To reduce participant burden, only the PAS was completed before and after each activity (*α* = 0.94–0.97).

At preprogram, participants completed a demographic questionnaire containing age, gender identity, race/ethnicity, and military service information. Preprogram physical activity was measured with the 9-item International Physical Activity Questionnaire–Self-Report Short Form (IPAQ-SF; Craig et al., 2003). IPAQ-SF items assess the frequency and duration of moderate and vigorous physical activity, walking, and sitting that participants have engaged in over the last 7 days. A categorization of *highly physically active* equates to at least 60 min of moderate intensity activity per day; *moderate* equates to at least 30 min of moderate intensity activity on most days; and *low* equates to less than *moderate* (For a more detailed scoring, see IPAQ Research Committee, 2005). At preprogram and 3-month follow-up, veterans self-reported concurrent treatments and interventions using a treatment utilization questionnaire developed for the study.

### Data analysis

All data were checked for plausibility and analyzed as intent-to-treat. Mann–Whitney *U*-tests, independent *t*-tests, and Chi-square tests were run to determine whether any preprogram differences existed between diagnostic groups (i.e., probable PTSD vs. no PTSD). Activity order and type were not examined in the current analyses because the parent study found no significant differences by these factors (Walter et al., 2021).

Multilevel models (MLMs) in SPSS 25 (IBM, Armonk, NY, United States) were used to examine changes in psychological outcomes based on PTSD diagnostic status. Longitudinal (i.e., preprogram, postprogram, 3-month follow-up) models were run for the PCL-5, PHQ-8, GAD-7, ISI, PAS, and NAS. Daily activity (i.e., pre- and postsession) models were run for the PHQ-4 and PAS. Models were built using a step-up, model-building process with respect to model fit and theory. Covariance matrices were chosen based on Akaike information criterion and the number of parameters specified. All final models relied on restricted maximum likelihood to account for missing data, which uses all available data to estimate missing values. Missing data were determined as missing at random in the parent study (Walter et al., 2021).

In longitudinal models, time was used categorically because there were qualitative differences between time points (i.e., an intervention was delivered between pre- and postprogram but not in the follow-up period; timeframe); thus, a random time slope could not be estimated as residuals cannot be calculated based on only one data point per time point. Although random time slopes could not be set, we used a random effect of intercept and a repeated effect of time [as a function of participant with an unstructured covariance matrix]. Fixed effects included time, concurrent psychiatric medication, and PTSD diagnostic status.

In daily activity models, time represented pre- to postsession for each activity. A random effect of time was calculated given that the qualitative difference across all time points was equivalent; time was crossed with day (continuous) and there were multiple observations per time point (i.e., pre and postsession). Based on this, random effects used a first-order autoregressive covariance structure and included intercept, pre- to postsession, day, and the crossed effect of pre- to postsession by day—all as a function of participant. Repeated effects used time as a function of participant by day with an unstructured covariance matrix. Fixed effects in activity session models included time, concurrent psychiatric medication, and PTSD diagnostic status. For fixed effects in both longitudinal and activity models, preprogram physical activity level was removed before final models due to lack of significance, and concurrent psychotherapy was not included due to high correlation with concurrent pharmacotherapy.

## Results

A total of 74 NVSSC participants were included in the analysis; 20 were classified as having a probable PTSD diagnosis at preprogram and 54 were not. Those in the probable PTSD group reported more severe preprogram depression (*p* < 0.001), generalized anxiety (*p* < 0.001), and negative affect (*p* = 0.026) scores, and they were more likely to report use of concurrent treatment (*p* < 0.001); there were no other significant demographic differences. For additional descriptive information, see Table 1. MLM estimated marginal means for outcomes by PTSD status can be found in Table 2, and unadjusted model results can be found in Appendix Table S1.

**Table 1:** Preprogram sample characteristics.

Characteristic	Total sample (*N* = 74)	Probable PTSD (*n* = 20)	No PTSD (*n* = 54)
	*n* (%)	*n* (%)	*n* (%)
Race/ethnicity^a^			
Asian or Native Hawaiian/Other Pacific Islander	2 (2.8)	–	–
Black or African American	7 (9.5)	–	–
Hispanic, Latino, or Spanish origin	6 (8.1)	–	–
Multiracial	7 (9.5)	–	–
White	50 (67.6)	–	–
Gender identity^b^			
Men	55 (74.3)	14 (70.0)	41 (75.9)
Women	19 (25.7)	6 (30.0)	13 (24.1)
Concurrent treatment^c^	51 (68.9)	20 (100.0)^***^	31 (57.4)^***^
Pharmacotherapy	33 (44.6)	14 (70.0)^**^	19 (35.2)^**^
Outpatient mental health care	45 (60.8)	19 (95.0)^***^	26 (48.1)^***^
Activity level^d^			
Low/inactive	12 (16.2)	0 (0)	12 (22.2)
Moderately active	24 (32.4)	10 (50.0)	14 (25.9)
Highly active	26 (35.1)	9 (45.0)	17 (31.5)
	*M* (*SD*)	*M* (*SD*)	*M* (*SD*)
Age, years	50.7 (11.8)	52.8 (11.9)	50.0 (11.7)
Days attended	4.9 (0.5)	5.0 (0.2)	4.9 (0.5)
Preprogram measures			
PTSD (PCL-5)	–	46.0 (12.2)	19.4 (14.9)
Depression (PHQ-8)	7.5 (5.5)	11.0 (6.2)^***^	6.2 (4.6)^***^
Anxiety (GAD-7)	6.6 (5.5)	10.6 (5.7)^***^	5.1 (4.6)^***^
Insomnia (ISI)	12.8 (7.6)	15.0 (6.1)	11.9 (8.0)
Positive affect (PAS)	34.5 (8.7)	33.4 (8.7)	34.9 (8.7)
Negative affect (NAS)	16.1 (7.3)	19.0 (9.1)^*^	14.9 (6.2)^*^

PTSD, posttraumatic stress disorder; PCL-5, PTSD Checklist for DSM-5; PHQ-8, 8-item Patient Health Questionnaire; GAD-7, 7-item Generalized Anxiety Disorder Scale; ISI, Insomnia Severity Index; PAS, Positive Affect Schedule; NAS, Negative Affect Schedule.

a Attempts were made to report race/ethnicity and rank data properly, but variables were combined to protect identity due to low cell counts and are not stratified by condition.

b Gender identity was self-reported. Sex at birth was not collected.

c Because concurrent depression treatment categories are not mutually exclusive, the sum across treatment categories is greater than 100%.

d Physical activity level data were calculated (see IPAQ Research Committee, 2005) from the self-report version of the International Physical Activity Questionnaire - Short Form.

^*^p < 0.05; ^**^p < 0.01; ^***^p < 0.001.

**Table 2:** Estimated marginal means at all time points by probable PTSD status.

Outcome and time point	Probable PTSD (*n* = 20) *M* (95% CI)	No PTSD (*n* = 54) *M* (95% CI)
PHQ-8		
Preprogram	9.6 [7.5, 11.7]	6.3 [4.9, 7.7]
Postprogram	5.2 [3.3, 7.0]	2.2 [1.0, 3.5]
3-month follow-up	11.4 [8.4, 14.3]	7.1 [5.2, 9.1]
GAD-7		
Preprogram	9.6 [7.4, 11.8]	5.0 [3.5, 6.4]
Postprogram	3.5 [1.8, 5.2]	1.9 [0.7, 3.0]
3-month follow-up	10.3 [7.7, 13.0]	5.6 [3.8, 7.3]
ISI		
Preprogram	13.6 [10.0, 17.2]	11.9 [9.5, 14.3]
Postprogram	10.4 [7.2, 13.7]	8.9 [6.7, 11.1]
3-month follow-up	13.2 [9.5, 16.9]	10.3 [7.8, 12.7]
PAS		
Preprogram	35.0 [31.1, 38.9]	34.6 [32.0, 37.2]
Postprogram	42.3 [39.0, 45.6]	43.0 [40.8, 45.3]
3-month follow-up	31.1 [26.3, 35.8]	35.1 [31.9, 38.3]
NAS		
Preprogram	17.1 [14.2, 20.1]	15.1 [13.2, 17.1]
Postprogram	15.3 [13.4, 17.3]	11.7 [10.3, 13.0]
3-month follow-up	16.8 [13.8, 19.8]	14.6 [12.6, 16.6]
PHQ-4		
Presession	2.3 [1.8, 2.9]	1.4 [1.0, 1.8]
Postsession	1.6 [1.1, 2.1]	0.6 [0.2, 1.0]
PAS		
Presession	38.2 [35.3, 41.1]	39.1 [36.9, 41.4]
Postsession	41.2 [38.3, 44.0]	41.6 [39.3, 43.9]

PTSD, posttraumatic stress disorder; PHQ-8, 8-item Patient Health Questionnaire; GAD-7, 7-item Generalized Anxiety Disorder Scale; ISI, Insomnia Severity Index; PAS, Positive Affect Schedule; NAS, Negative Affect Schedule; PHQ-4, 4-item Patient Health Questionnaire. Estimated marginal means were derived from multilevel models that controlled for concurrent psychiatric medication use and probable PTSD status. Day of program was also controlled for in session analyses. PTSD symptom severity was only examined as an outcome among those with probable PTSD and is thus not displayed in this comparative table.

### Psychological symptoms

For those with probable PTSD at preprogram, PCL-5 scores significantly decreased from pre- to postprogram (*MD* = −23.76, *p* < 0.001) but then rebounded to preprogram scores at 3-month follow-up (postprogram to 3-month, *MD* = 22.18, *p* = 0.005). Specifically, PTSD severity scores were 43.15 at preprogram, 19.39 at postprogram, and 41.57 at 3-month follow-up when controlling for concurrent psychiatric medication. Change in PTSD symptom severity was statistically significant and reliable; however, it did not exceed the suggested cutoff for clinical significance on the PCL-5 (28 points; Marx et al., 2022). PTSD scores were not compared between diagnostic groups because only one group met probable PTSD criteria based on a Criterion A traumatic event. For complete longitudinal program results, see Table 3.

**Table 3:** Mean differences from estimated marginal means of final multilevel models examining psychological outcomes.

Predictor	*MD*	95% CI	*p*
PCL-5			
Time			
Pre- to postprogram	−23.76	[−31.94, −15.57]	**<0.001**
Postprogram to 3-month follow-up	22.18	[6.87, 37.50]	**0.005**
Preprogram to 3-month follow-up	−1.58	[−10.29, 7.14]	1.00
Pharmacotherapy	8.34	[−3.34, 20.03]	0.150
Time × Pharmacotherapy			
Time (pre-post) × Pharmacotherapy	−3.91	[−17.96, 10.14]	0.564
Time (post-3 month) × Pharmacotherapy	0.76	[−25.49, 27.01]	0.952
Time (pre-3 month) × Pharmacotherapy	−3.15	[−18.05, 11.74]	0.657
PHQ-8			
Time			
Pre- to postprogram	−4.23	[−5.59, −2.88]	**<0.001**
Postprogram to 3-month follow-up	5.56	[3.43, 7.68]	**<0.001**
Preprogram to 3-month follow-up	1.32	[−0.51, 3.16]	0.204
PTSD diagnosis	3.49	[1.28, 5.71]	**0.003**
Time × PTSD diagnosis			
Time (pre-post) × PTSD diagnosis	−0.43	[−2.94, 2.09]	0.736
Time (post-3 month) × PTSD diagnosis	1.32	[−2.58, 5.22]	0.498
Time (pre-3 month) × PTSD diagnosis	0.89	[−2.46, 4.25]	0.596
Pharmacotherapy	3.28	[1.23, 5.33]	**0.002**
Time × Pharmacotherapy			
Time (pre-post) × Pharmacotherapy	−3.00	[−5.35, −0.65]	**0.013**
Time (post-3 month) × Pharmacotherapy	0.84	[−2.78, 4.45]	0.642
Time (pre-3 month) × Pharmacotherapy	−2.16	[−5.26, 0.93]	0.166
GAD			
Time			
Pre- to postprogram	−4.61	[−6.14, −3.08]	**<0.001**
Postprogram to 3-month follow-up	5.26	[3.36, 7.17]	**<0.001**
Preprogram to 3-month follow-up	0.66	[−0.84, 2.16]	0.628
PTSD diagnosis	3.66	[1.57, 5.76]	**0.001**
Time × PTSD diagnosis			
Time (pre-post) × PTSD diagnosis	−3.07	[−5.90, −0.24]	**0.034**
Time (post-3 month) × PTSD Diagnosis	3.12	[−0.38, 6.61]	0.079
Time (pre-3 month) × PTSD Diagnosis	0.05	[−2.69, 2.79]	0.971
Pharmacotherapy	2.98	[1.05, 4.92]	**0.003**
Time × Pharmacotherapy			
Time (pre-post) × Pharmacotherapy	−1.67	[−4.29, 0.96]	0.209
Time (post-3 month) × Pharmacotherapy	1.55	[−1.68, 4.79]	0.340
Time (pre-3 month) × Pharmacotherapy	−0.11	[−2.64, 2.41]	0.929
ISI			
Time			
Pre- to postprogram	−3.08	[−4.63, −1.53]	**<0.001**
Postprogram to 3-month follow-up	2.05	[−0.11, 4.21]	0.066
Preprogram to 3-month follow-up	−1.03	[−2.70, 0.65]	0.327
PTSD diagnosis	2.05	[−1.87, 5.97]	0.300
Time × PTSD diagnosis			
Time (pre-post) × PTSD diagnosis	−0.18	[−3.03, 2.68]	0.902
Time (post-3 month) × PTSD diagnosis	1.38	[−2.60, 5.35]	0.490
Time (pre-3 month) × PTSD Diagnosis	1.20	[−1.88, 4.28]	0.437
Pharmacotherapy	4.79	[1.16, 8.42]	**0.011**
Time × Pharmacotherapy			
Time (pre-post) × Pharmacotherapy	−1.31	[−3.99, 1.38]	0.334
Time (post-3 month) × Pharmacotherapy	1.19	[−2.53, 4.90]	0.525
Time (pre-3 month) × Pharmacotherapy	−0.12	[−2.99, 2.75]	0.933
PAS			
Time			
Pre- to postprogram	7.88	[5.09, 10.66]	**<0.001**
Postprogram to 3-month follow-up	−9.62	[−13.01, −6.22]	0.390
Preprogram to 3-month follow-up	−1.74	[−4.80, 1.32]	0.390
PTSD diagnosis	−1.44	[−5.20, 2.32]	0.446
Time × PTSD diagnosis			
Time (pre-post) × PTSD diagnosis	−1.13	[−6.29, 4.02]	0.661
Time (post-3 month) × PTSD Diagnosis	−3.34	[−9.57, 2.88]	0.286
Time (pre-3 month) × PTSD diagnosis	−4.48	[−10.07, 1.11]	0.114
Pharmacotherapy	−3.32	[−6.80, 0.16]	0.061
Time × Pharmacotherapy			
Time (pre-post) × Pharmacotherapy	7.80	[2.99, 12.62]	**0.002**
Time (post-3 month) × Pharmacotherapy	−5.23	[−11.01, 0.56]	0.076
Time (pre-3 month) × Pharmacotherapy	2.57	[−2.59, 7.74]	0.322
NAS			
Time			
Pre- to postprogram	−2.62	[−4.41, −0.83]	**0.003**
Postprogram to 3-month follow-up	2.21	[0.40, 4.02]	**0.014**
Preprogram to 3-month follow-up	−0.41	[−2.73, 1.92]	1.00
PTSD diagnosis	2.62	[0.05, 5.18]	**0.046**
Time × PTSD diagnosis			
Time (pre-post) × PTSD diagnosis	1.66	[−1.66, 4.98]	0.321
Time (post-3 month) × PTSD diagnosis	−1.51	[−4.81, 1.80]	0.364
Time (pre-3 month) × PTSD Diagnosis	0.16	[−4.13, 4.44]	0.942
Pharmacotherapy	3.78	[1.41, 6.15]	**0.002**
Time × Pharmacotherapy			
Time (pre-post) × Pharmacotherapy	−4.00	[−7.09, −0.91]	**0.012**
Time (post-3 month) × Pharmacotherapy	4.98	[1.91, 8.05]	**0.002**
Time (pre-3 month) × Pharmacotherapy	0.98	[−2.97, 4.93]	0.621

MD, mean difference; PTSD, posttraumatic stress disorder; PCL-5, PTSD Checklist for DSM-5; PHQ-8, 8-item Patient Health Questionnaire; GAD-7, 7-item Generalized Anxiety Disorder; ISI, Insomnia Severity Index; PAS, Positive Affect Schedule; NAS, Negative Affect Schedule. Bolding indicates statistical significance. Positive probable PTSD diagnosis is the comparator for the PTSD diagnosis MDs; concurrent pharmacotherapy is the comparator for the Pharmacotherapy MDs.

Across participants, PHQ-8 scores significantly decreased from pre- to postprogram (*MD* = −4.23, *p* < 0.001) then returned to preprogram scores during the 3-month follow-up period (*MD* = 5.56, *p* < 0.001) after controlling for concurrent treatment and PTSD diagnostic status. Change in depression severity was statistically significant but did not reach the level of clinical significance on the PHQ-8 (e.g., 5 points; Schneider et al., 2020). Specifically, depression severity scores decreased from 7.94 to 3.70 points then returned to 9.26 points. The interaction addressing the main study aim was not significant (Time × PTSD Diagnosis, *p* = 0.792), suggesting that probable PTSD diagnosis did not effect change in depression severity over time.

GAD-7 scores significantly improved from pre- to postprogram (*MD* = −4.61, *p* < 0.001) and reverted to preprogram scores at 3-month follow-up (*MD* = 5.26, *p* < 0.001) when accounting for other factors. Generalized anxiety scores decreased from 7.29 to 2.69 points then increased to 7.95 points in the follow-up period. Although the overall Time × PTSD Diagnosis interaction was not significant (i.e., spanning preprogram to 3-month follow-up; *p* = 0.093), inspection of simple effects showed a statistically significant difference between groups from pre- to postprogram (*MD* = −3.07, *p* = 0.034). Specifically, those with probable PTSD decreased 3.07 points more than those without at postprogram. Generalized anxiety was the single outcome demonstrating a significant between-group difference over time. This difference was statistically significant but not at the level of clinical significance on the GAD-7 (4 points; Toussaint et al., 2020).

PHQ-4 scores were used to assess depression and anxiety before and after each daily activity. These scores significantly improved (*MD* = −0.75, *p* < 0.001) over the course of the day. However, there were no differences between PTSD diagnostic groups across time (*p* = 0.961; see Table 4).

**Table 4:** Mean differences from estimated marginal means of final multilevel models examining activity session outcomes.

Predictor	*MD*	95% CI	*p*
PHQ-4			
Time (pre- to postsession)	−0.75	[−1.01, −0.49]	**<0.001**
Day	−0.11	[−0.28, 0.06]	0.193
Time × Day	−0.02	[−0.17, 0.13]	0.797
PTSD diagnosis	0.99	[0.44, 1.55]	**0.001**
Time × PTSD diagnosis	0.01	[−0.52, 0.55]	0.961
Pharmacotherapy	1.00	[0.48, 1.51]	**<0.001**
Time × Pharmacotherapy	−0.64	[−1.14, −0.14]	**0.012**
PAS			
Time (pre- to postsession)	2.71	[1.35, 4.06]	**<0.001**
Day	0.43	[−0.48, 1.35]	0.348
Time × Day	−0.02	[−0.71, 0.67]	0.947
PTSD diagnosis	−0.68	[−3.29, 1.94]	0.612
Time × PTSD diagnosis	0.46	[−2.02, 2.93]	0.714
Pharmacotherapy	−2.21	[−4.63, 0.21]	0.073
Time × Pharmacotherapy	3.04	[0.76, 5.33]	**0.010**

MD, mean difference; CI, confidence interval; PHQ-4, 4-item Patient Health Questionnaire; PTSD, posttraumatic stress disorder; PAS, Positive Affect Schedule. Bolding indicates statistical significance. Postsession is the comparator for the time variable MDs; positive probable PTSD diagnosis is the comparator for the PTSD diagnosis; concurrent pharmacotherapy is the comparator for the Pharmacotherapy MDs.

Self-reported insomnia symptoms significantly improved during the program, as indicated by changes in ISI scores from pre- to postprogram (*MD* = −3.08, *p* < 0.001). During the follow-up period, scores returned to preprogram levels (*MD* = 2.05, *p* = 0.066; preprogram to 3-month *p* = 0.327). Changes in insomnia symptoms over time did not vary by probable PTSD diagnostic status (*p* = 0.721).

### Affect

Overall, positive affect significantly improved from pre- to postprogram (*MD* = 7.88, *p* < 0.001), but not during the follow-up period (*MD* = −9.62, *p* < 0.001). PAS scores increased from 34.81 to 42.69 points during the program before returning to 33.07 at 3-month follow-up. There was no significant difference over time by probable PTSD diagnostic status (*p* = 0.281). Daily activity scores followed a similar pattern, where PAS scores improved from pre- to postsession (*MD* = 2.71, *p* < 0.001) but did not differ over time by PTSD status (*p* = 0.714).

Negative affect significantly decreased among participants from pre- to postprogram (*MD* = −2.62, *p* = 0.003), and like other outcomes, scores returned to preprogram scores by the 3-month follow-up (*MD* = 2.21, *p* = 0.014). On average, NAS scores decreased from 16.12 to 13.50 points before increasing to 15.72 points at 3-month follow-up. Change in NAS scores over time was not influenced by probable PTSD diagnostic status (*p* = 0.456).

## Discussion

Given that PTSD is one of the most common psychological diagnoses among veterans and service members (e.g., Fulton et al., 2015; Thomas et al., 2017) and that recreational and adaptive sports programs are often available for these populations, it is critical to explore how these programs impact psychological health among veterans with PTSD. The current study compared psychological outcomes following NVSSC program participation among veterans with and without probable PTSD. Overall, veterans with probable PTSD reported similar psychological benefits to the NVSSC program compared to those without PTSD when added to their current treatment plan. Results from this study demonstrated that U.S. veterans with and without probable PTSD reported significant improvements in depression, generalized anxiety, insomnia, positive affect, and negative affect immediately following participation in the week-long NVSSC program, as well as improvements in depression/anxiety and positive affect immediately following a session activity. Veterans with probable PTSD also reported significant reductions in PTSD symptom severity over the course of the program, which were reliable and statistically significant. However, the change in PTSD symptom severity approached, but did not exceed, suggested thresholds for clinical significance (Marx et al., 2022). In general, the only significant difference in longitudinal outcomes between veterans with and without PTSD was for generalized anxiety (observed from pre- to postprogram), although this finding was not clinically meaningful (Toussaint et al., 2020). This finding may be explained by the fact that on average, veterans with PTSD had higher generalized anxiety severity scores compared to those without PTSD. Thus, veterans with probable PTSD had greater room for improvement.

Study findings reflect the current literature, which suggests that recreational and adaptive sports programs have positive short-term psychological outcomes among military populations with PTSD (e.g., Davis-Berman et al., 2018; Forsyth et al., 2020). Consistent with these findings, Walter et al. (2019) showed that outcomes for service members with and without probable PTSD were comparable directly following 6 weeks of surf therapy (i.e., at postprogram). These findings were later extended to demonstrate similar outcomes between surf therapy participants with both major depressive disorder and PTSD (Otis et al., 2020) and among hike therapy participants (Otis et al., in press). Overall, the benefits of recreational and adaptive sports programs seem to be broadly beneficial for military populations with and without PTSD.

Study results also replicate several studies demonstrating that positive outcomes for physically based interventions may not be sustained in the weeks and months following participation (e.g., Townsend et al., 2018; Walter et al., 2021; Hooker et al., 2022). Across all psychological outcomes and both diagnostic groups, scores rebounded to preprogram levels at 3-month follow-up, suggesting that gains were not maintained. Walter et al. (2023) used similar assessment methodology to this study to evaluate the duration of psychological effects following a randomized trial of surf and hike therapy. The Surf and Hike Therapy programs were conducted locally on a weekly basis for 6 consecutive weeks whereas the NVSSC was a week-long program in San Diego that typically required participants to travel. Unlike findings from the current study that showed a rebound in symptoms at the 3-month follow-up, results at 3 months following Surf and Hike Therapy demonstrated continued gains in depression outcomes for active duty service member participants (Walter et al., 2023). While these findings cannot be directly compared, together they may suggest that programs with longer duration, more regular sessions, occur locally, or provide opportunities for continued engagement following the program period may facilitate sustained psychological improvements (e.g., Babyak et al., 2000; Mota-Pereira et al., 2013). Additional research is needed to identify program factors (e.g., length of program, frequency of sessions, proximity of location) that are most beneficial and predictors of symptom regression so that researchers and clinicians can develop optimal recreational and adaptive sports programs that provide lasting psychological benefit.

Demonstrating positive mental health effects of NVSSC program participation for veterans with PTSD is important because military populations with this disorder can be both difficult to treat (e.g., Steenkamp et al., 2015; Resick et al., 2017) and reluctant to seek treatment due to factors such as stigma (e.g., Hoge et al., 2004; Pietrzak et al., 2009; Kulesza et al., 2015; Sharp et al., 2015), negative beliefs about care, and logistical issues (e.g., Stecker et al., 2013; Hoge et al., 2014). Adaptive and recreational treatments—in combination with traditional interventions or as a first step in engaging in therapeutic options—may serve an important role in the treatment “package” offered to those with PTSD. These programs may serve a unique function in this population because they are available to veterans and service members with a variety of physical and psychological diagnoses, may not trigger the same level of stigma as traditional therapies, and promote socialization and other positive mental health outcomes (e.g., Davis-Berman et al., 2018; Greer and Vin-Raviv, 2019; Forsyth et al., 2020). Further, our findings add to a growing body of literature indicating there appears to be both global and disorder-specific effects for recreational and adaptive sports interventions (Walter et al., 2019; Otis et al., 2020, in press). Allowing these programs to be inclusivity oriented may ensure that populations at greatest need for these interventions are able to receive them.

This work has several important limitations and findings should be interpreted accordingly. The study used naturalistic design with groups created *a posteriori* to address the study aim—determining whether NVSSC program outcomes differed between veterans with and without probable PTSD—and was not conducted as an experimental design or randomized controlled trial. As such, it is impossible to know whether our longitudinal findings are directly the result of NVSSC, other interventions or confounds, or a combination of factors. Similarly, concurrent outpatient mental health treatment was not included in analyses due to lack of variability (95% of the probable PTSD group); instead, we used concurrent psychiatric medication in the models, which showed greater variability (70% of the probable PTSD group). Given the overlap between preprogram concurrent psychiatric medication use and PTSD diagnostic status, we cannot separate the effects of each and acknowledge the question of whether such programs serve as a standalone or adjunctive intervention approach. This study did not measure participants’ levels of physical activity during the follow-up period, so we were unable to determine whether those who continued to engage in physical activity were more likely to retain the psychological benefits following the NVSSC. Physical activity data collected *via* the IPAQ-SF were only collected at preprogram as the measure was initially developed as a survey tool rather than as an instrument to evaluate change (e.g., Craig et al., 2003). Although outcome data exhibited a quadratic pattern, time was unable to be examined quadratically due to uneven spacing between assessment points. The subsample of veterans with probable PTSD was small (*n* = 20) and may impact generalizability and representativeness of the sample. Probable PTSD diagnosis was also determined by applying diagnostic scoring criteria to a self-report measure (i.e., PCL-5). Ideally, a clinician-administered interview would be used to establish a PTSD diagnosis. However, the PCL-5 is a well-validated assessment for this purpose and widely used in military populations (Bovin et al., 2016; Forkus et al., 2022). The remainder of outcomes were assessed with self-report measures, which may be subject to bias. Finally, postprogram assessments were captured on the last day of the program, immediately following the end of the last session, which may have conflated program and activity effects.

There are also several study strengths that add to the current body of literature examining recreational and adaptive sports programs among veterans and service members with PTSD. Although many of these programs exist, there are limited data evaluating their effectiveness for veterans and service members with PTSD. The current study examined NVSSC program outcomes for veterans with probable PTSD in comparison to those without to determine whether there are disorder-specific or more global psychological effects. The naturalistic design improves external validity by evaluating the program as it exists in the “real world.” Assessments were administered at various time points, including pre- and postsession, as well as preprogram, postprogram, and 3-month follow-up, allowing for an examination of immediate, short-term, and longer-term program outcomes. Lastly, well-validated self-report assessments and statistical analyses intended for longitudinal data were used to maximize confidence in the data and associated findings.

This study compared NVSSC outcomes between veteran participants with and without probable PTSD. Study results support the psychological benefits of the NVSSC for veterans with and without probable PTSD and contribute to the research evaluating recreational and adaptive sports programs. Along with the parent study, our data show benefits over the course of the NVSSC for veterans with and without probable PTSD that were not maintained in the follow-up period, suggesting that ongoing engagement and programs that occur more frequently or that are local to veterans may help sustain gains (e.g., Walter et al., 2021). However, our findings also highlight areas for continued research to ensure that the benefits of these programs, which can improve psychological health and may reduce stigma, are sustained over time. Additional research is needed to determine what factors hinder or protect against relapse during the follow-up period, and then adapt programs to include prevention methods. This is not uncommon. Many evidence-based psychological interventions include “relapse prevention” strategies (e.g., Resick et al., 2016; Foa et al., 2019), and recreational interventions may benefit from the same strategy. Available evidence to date suggests that recreational and adaptive sports interventions may offer benefits to veterans with PTSD, but lingering questions remain regarding whether these interventions fit into the broader PTSD treatment guidelines (e.g., standalone vs. adjunctive therapy) as well as how to ensure benefits are sustained over time.

## Data availability statement

The dataset presented in this article is not readily available because of security protocols and privacy regulations, but they may be made available on reasonable request by the VA San Diego Healthcare System or by contacting the corresponding author to facilitate the request. Requests to access the datasets should be directed to KW, kristen.h.walter.civ@health.mil.

## Ethics statement

The studies involving human participants were reviewed and approved by the VA San Diego Healthcare System Institutional Review Board, protocol number H170050. The patients/participants provided their written informed consent to participate in this study.

## Author contributions

KW: secured funding, conceptualization, methodology, investigation, resources, writing—original draft, supervision, and project administration. NO: conceptualization, methodology, formal analysis, writing—original draft, data curation, resources, and project management. MH and KO: investigation, supervision, resources, recruitment, and project administration. LG: investigation, data curation, manuscript conceptualization, writing—original draft, supervision. All authors reviewed, edited, and approved the final manuscript.

## Funding

This work was supported in part by the U.S. Navy Bureau of Medicine and Surgery (BUMED) under work unit no. N1600. BUMED had no role in the study design, collection, analysis, or interpretation of the data, writing the manuscript, or the decision to submit the paper for publication.

## Conflict of interest

NO and LG are employed by Leidos, Inc.

The remaining authors declare that the research was conducted in the absence of any commercial or financial relationships that could be construed as a potential conflict of interest.

## Publisher’s note

All claims expressed in this article are solely those of the authors and do not necessarily represent those of their affiliated organizations, or those of the publisher, the editors and the reviewers. Any product that may be evaluated in this article, or claim that may be made by its manufacturer, is not guaranteed or endorsed by the publisher.
