# Supplementary material for: The effectiveness of the National Veterans Summer Sports Clinic for veterans with probable posttraumatic stress disorder
Source: Front Psychol. 2023 Jul 10;14:1207633. doi: 10.3389/fpsyg.2023.1207633 (PMC10363688; doi:10.3389/fpsyg.2023.1207633)
Supplement: Supplementary file 1 [file Table_1.DOCX]

Supplementary Material

The Effectiveness of the National Veterans Summer Sports Clinic for Veterans with Probable PTSD

**Kristen H. Walter^*^, Nicholas P. Otis, Michal Kalli Hose, Kathleen M. Ober, and Lisa H. Glassman**

*** Correspondence:** Kristen H. Walter: [kristen.h.walter.civ@health.mil](mailto:kristen.h.walter.civ@health.mil)

# Supplementary Table

**TABLE S1** Estimates from unadjusted multilevel models of time on outcome (*N* = 74)

| Time point | B | 95% CI | *p* |
| --- | --- | --- | --- |
| PCL-5 | | | |
| Intercept (preprogram) | 46.00 | [40.31, 51.69] | **<.001** |
| Pre- to postprogram | −25.85 | [−32.06, −19.64] | **<.001** |
| Postprogram to 3-month follow-up | 23.11 | [10.71, 35.51] | **<.001** |
| Preprogram to 3-month follow-up | −2.74 | [−8.83, 3.36] | .356 |
| PHQ-8 | | | |
| Intercept (preprogram) | 7.47 | [6.19, 8.74] | **<.001** |
| Pre- to postprogram | −4.27 | [−5.59, −2.88] | **<.001** |
| Postprogram to 3-month follow-up | 5.68 | [4.00, 7.35] | **<.001** |
| Preprogram to 3-month follow-up | 1.40 | [−0.14, 2.94] | .081 |
| GAD | | | |
| Intercept (preprogram) | 6.54 | [5.27, 7.80] | **<.001** |
| Pre- to postprogram | −3.91 | [−5.11, −2.71] | **<.001** |
| Postprogram to 3-month follow-up | 4.65 | [3.03, 6.26] | **<.001** |
| Preprogram to 3-month follow-up | 0.74 | [−0.32, 1.80] | .170 |
| ISI | | | |
| Intercept (preprogram) | 12.27 | [10.47, 14.08] | **<.001** |
| Pre- to postprogram | −2.90 | [−4.04, −1.77] | **<.001** |
| Postprogram to 3-month follow-up | 1.38 | [0.16, –0.40] | .066 |
| Preprogram to 3-month follow-up | −1.52 | [−2.78, –0.26] | **.019** |
| PAS | | | |
| Intercept (preprogram) | 34.67 | [32.62, 36.72] | **<.001** |
| Pre- to postprogram | 7.84 | [5.70, 9.97] | **<.001** |
| Postprogram to 3-month follow-up | −8.88 | [−11.74, −6.02] | **<.001** |
| Preprogram to 3-month follow-up | −1.04 | [−3.28, 1.20] | .357 |
| NAS | | | |
| Intercept (preprogram) | 15.97 | [14.27, 17.67] | **<.001** |
| Pre- to postprogram | −3.12 | [−4.59, −1.66] | **<.001** |
| Postprogram to 3-month follow-up | 2.33 | [0.73, 3.92] | **.003** |
| Preprogram to 3-month follow-up | −0.79 | [−2.46, 0.87] | .345 |
| PHQ-4 (session) | | | |
| Presession | 1.62 | [1.29, 1.96] | **<.001** |
| Pre- to postsession | –0.71 | [–0.93, –0.49] | **<.001** |
| PAS (session) | | | |
| Presession | 39.02 | [36.95, 41.08] | **<.001** |
| Pre- to postsession | 2.46 | [1.28, 3.64] | **<.001** |

CI, confidence interval; PCL-5, PTSD Checklist for DSM-5; PHQ-8, 8-item Patient Health Questionnaire; GAD-7, 7-item Generalized Anxiety Disorder; ISI, Insomnia Severity Index, PAS, Positive Affect Schedule; NAS, Negative Affect Schedule; PHQ-4, 4-item Patient Health Questionnaire.

Bolding indicates statistical significance.

In session models, time represented pre- to postactivity, and was crossed with day (continuous). For interpretability, only pre- to postactivity is reported here.
